# Supplementary material for: Efficient transgenesis and annotated genome sequence of the regenerative flatworm model Macrostomum lignano
Source: Nat Commun. 2017 Dec 14;8:2120. doi: 10.1038/s41467-017-02214-8 (PMC5730564; doi:10.1038/s41467-017-02214-8)
Supplement: Supplementary file 1 — Supplementary Information [file 41467_2017_2214_MOESM1_ESM.pdf]

Supplementary Table 1 | Effect of microinjection treatment on egg hatching

Treatment	No. treated eggs	No. survived injection (%)	No. hatched (%)
Transferring only	197	-	164 (83)
Microinjection H_20	147	113 (77)	108 (73)
Microinjection Alexa555	194	135 (70)	102 (53)

Supplementary Table 2 | Effect of irradiation treatment on egg survival

Irradiation dose (Gy)	No. irradiated eggs	No. hatched eggs (%)
10	33	3 (10)
5	34	30 (88)
2.5	35	33 (94)

Supplementary Table 3 | Genomic libraries generated in this study

Library	Technology	Insert	Reads[a]	Bases[a]	Accession
ML_SHG_1	454	300	1,251,825	332,023,742	SRX2866468
ML_SHG_2	454	300	1,961,618	633,169,545	SRX2866469
ML_SHG_3	454	300	2,215,605	728,249,532	SRX2866470
ML_SHG_4	454	300	1,080,486	350,450,106	SRX2866471
ML_SHG_5	454	300	1,227,790	424,947,781	SRX2866472
ML_3KB_1	454	3 kb	219,785	43,607,257	SRX2866473
ML_3KB_2	454	3 kb	234,763	46,858,248	SRX2866474
ML_3KB_3	454	3 kb	1,250,995	259,012,232	SRX2866475
ML_8KB_1	454	8 kb	1,700,452	299,532,504	SRX2866466
ML_8KB_2	454	8 kb	266,899	34,879,000	SRX2866467
ML_20KB_1	454	20 kb	349,619	67,963,897	SRX2866478
ML_20KB_2	454	20 kb	332,274	62,235,772	SRX2866479
ML_20KB_3	454	20 kb	916,967	187,484,600	SRX2866476
ML_20KB_4	454	20 kb	908,502	185,998,230	SRX2866477
HUB1_180	Illumina	130	447,533,260	44,294,490,982	SRX2866482
HUB1_300	Illumina	230	404,286,320	40,385,023,072	SRX2866483
DV1-400-1	Illumina	310	37,574,290	3,904,917,967	SRX2866480
DV1-400-2	Illumina	340	22,062,242	2,290,449,054	SRX2866481
DV1-600-1	Illumina	500	13,201,262	1,371,998,967	SRX2866484

DV1-600-2	Illumina	500	13,917,788	1,446,369,219	SRX2866485
DV1-3kb-1	Illumina	2.7 kb	47,469,690	4,803,681,174	SRX2866494
HUB1-5_4kb	Illumina	2.7 kb	67,616,074	6,540,147,915	SRX2866493
DV1-3kb-2	Illumina	3.1 kb	137,109,462	13,628,485,913	SRX2866492
DV1-6kb-1	Illumina	5.3 kb	42,907,318	4,335,393,561	SRX2866491
HUB1-3_6kb	Illumina	6.3 kb	12,574,102	1,202,742,615	SRX2866490
HUB1-3_7kb	Illumina	6.8 kb	13,847,722	1,337,266,809	SRX2866489
DV1-9kb-1	Illumina	7.8 kb	55,306,018	5,621,758,481	SRX2866488
HUB1-4_10kb	Illumina	12.4 kb	30,165,426	2,948,889,118	SRX2866487
HUB1-4_9kb	Illumina	13 kb	14,677,860	1,431,942,626	SRX2866486
**TOTAL**				**139 Gb, 185x**	

[a] After adapters and quality trimming.

Supplementary Table 4 | Repeats in Mlig_3_7 genome assembly

Repeat group	% of the genome
DNA transposons	3.05
non-LTR retrotransposons (LINE)	1.66
LTR retrotransposons	20.69
SINEs	0.39
Low complexity and simple repeats	1.34
Tandem repeats	13.12
Other repeats identified by Red	10.05
**Total repeats**	**50.30**

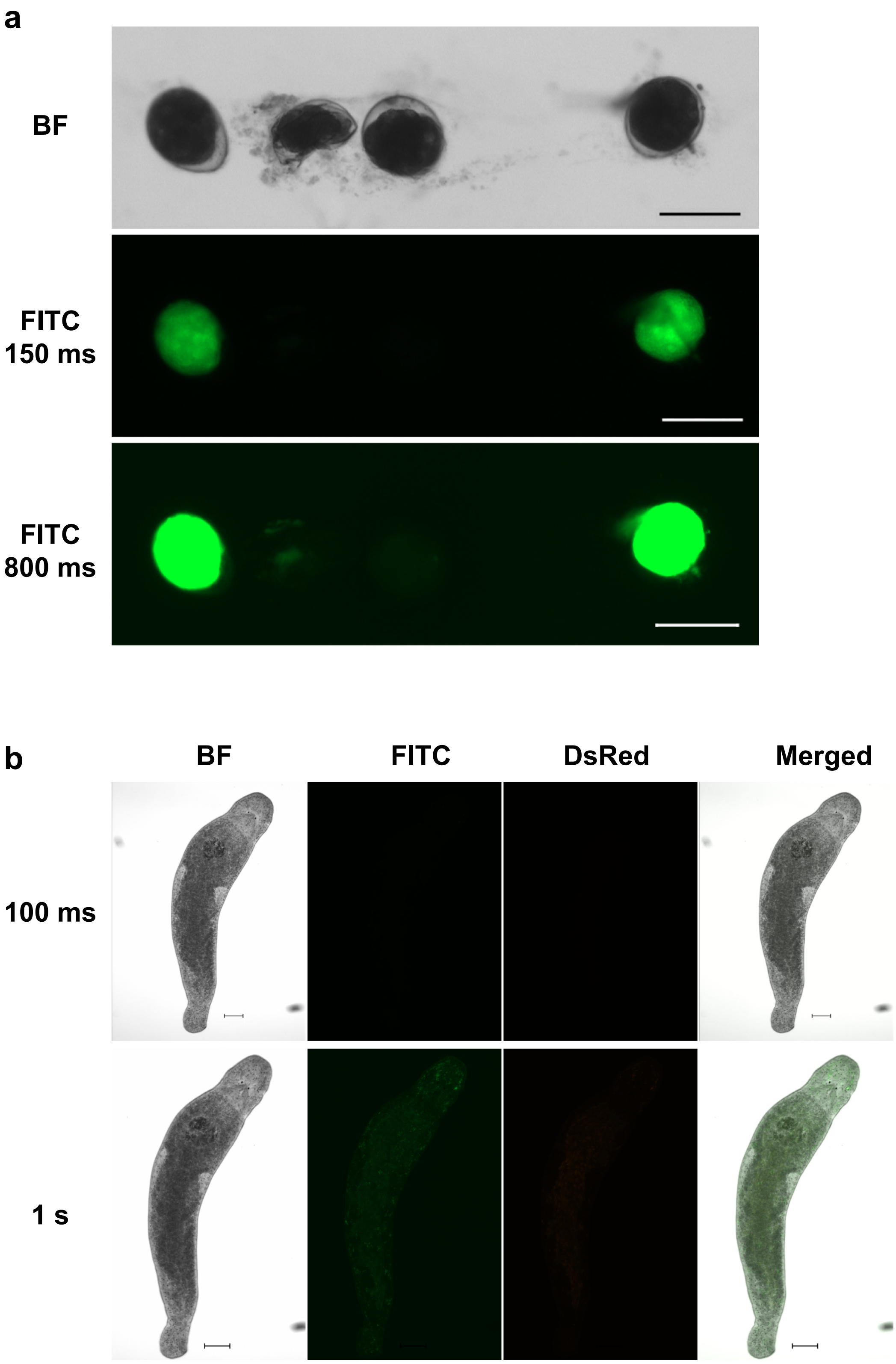

Supplementary Figure 1 | Levels of autofluorescence in *M. lignano* embryos and adult animals. (**a**) Injection of *gfp* mRNA into embryos. From left to right: correctly injected embryo, embryo destroyed upon microinjection; non-injected embryo, correctly injected embryo. BF – bright-field; FITC 150 ms and FITC 800 ms – FITC channel, exposure for 100 ms and 800 ms respectively. (**b**). Adult non-transgenic animals, DV1 strain. BF – bright-field; FITC – FITC channel, DsRed – DsRed channel, Merged – merged image from the three channels. 100 ms and 1 s – exposure times in FITC and DsRed channels. Scale bars are 100 µm.

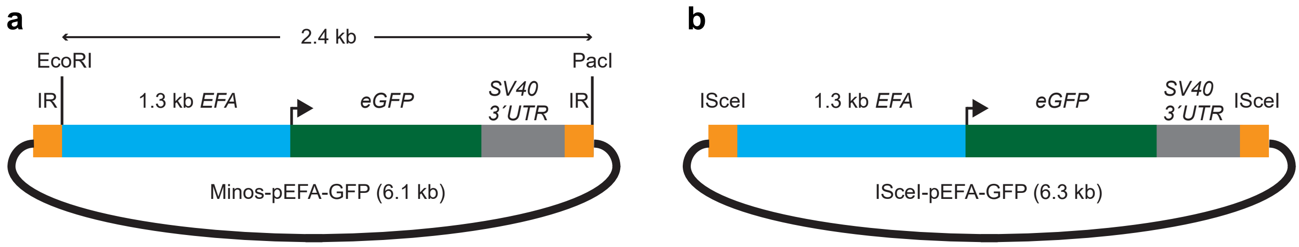

c

Injection	No. injected eggs	No. detected GFP expression (%)	No. germline transmission (line name)
Minos-p*EFA::GFP* with *Minos* mRNA	93	9 (9.7)	1 (HUB1)
	73	3 (4.4)	1 (HUB3)
	181	11 (6.1)	1 (HUB5)
	247	19 (7.6)	0
Minos-p*EFA::GFP* without *Minos* mRNA	86	6 (6.9)	0
	150	8 (5.3)	0
ISceI-p*EFA::GFP* with ISceI protein	80	4 (5.0)	0
	65	2 (3.1)	0
	73	6 (8.2)	1 (HUB4)
	83	6 (7.2)	0
	101	3 (3.6)	0
	120	5 (4.2)	0
ISceI-p*Efa::GFP* without ISceI protein	80	7 (10)	1 (HUB2)
	156	8 (5.1)	0

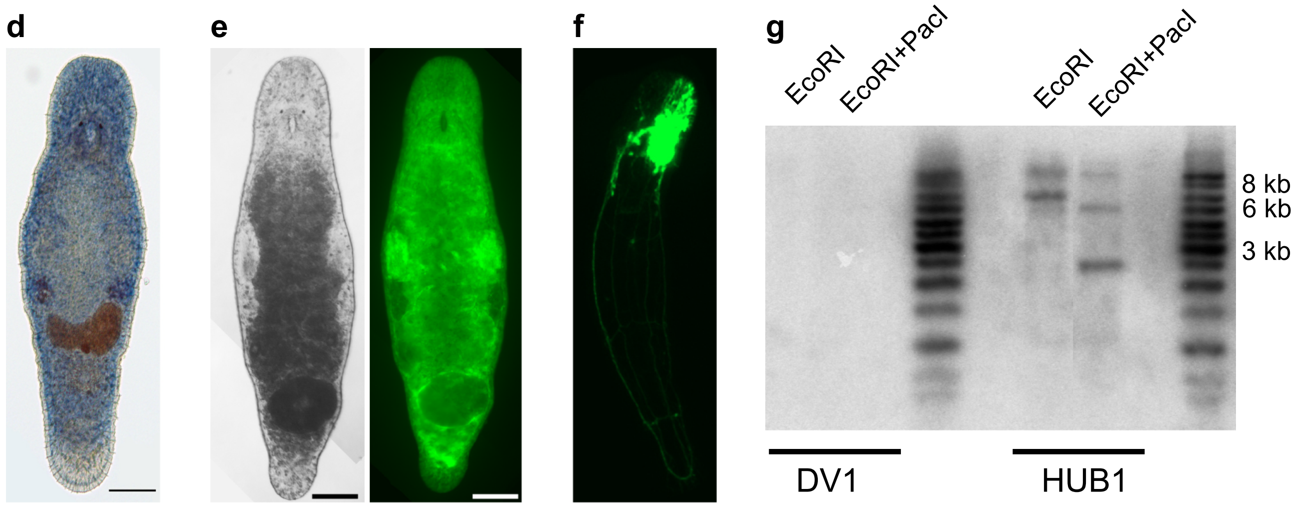

Supplementary Figure 2 | Initial transgenesis attempts in *M. lignano* using transcriptional fusion of the Elongation factor alpha promoter sequence with *eGFP*. (**a**) *Minos* transposon-based construct. (**b**) Meganuclease *I-SceI* based construct. (**c**) Efficiency of transgenesis with different injection combinations, without irradiation. (**d**) Whole mount *in situ* hybridization expression pattern of *EFA* gene. (**e**) Stable expression in HUB1 transgenic animal, bright-field and FITC channels. (**f**) Transient expression of p*EFA::eGFP* 3 months after hatching. Note that most tissue was replaced by non-fluorescent neoblasts. Due to the low turnover of the nervous system tissue the fluorescence remained in the brain and the nerve cords. (**g**) Southern blot analysis of HUB1 line demonstrating presence of several copies of the transgene. DV1 – original wild-type line used to create HUB1.

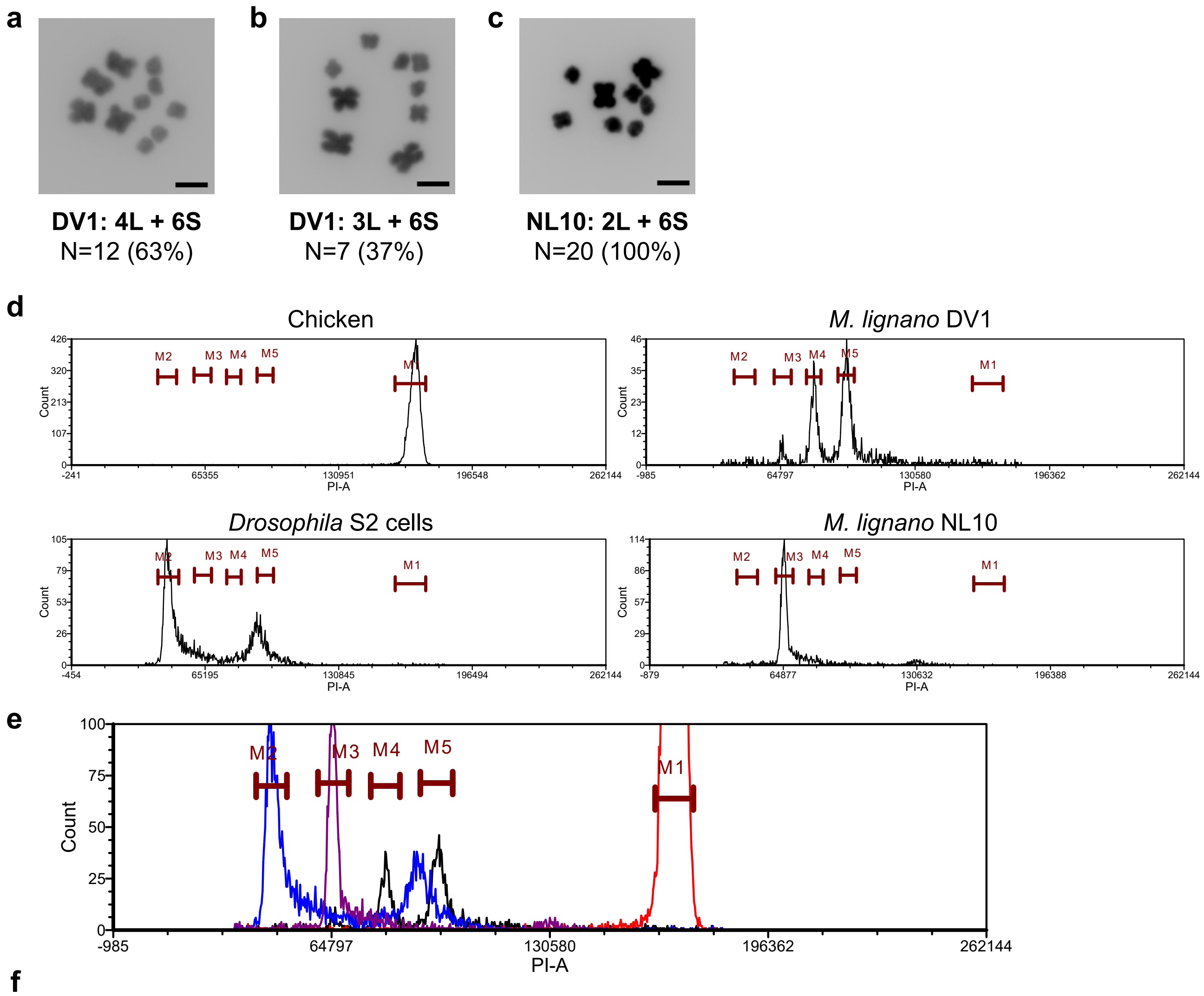

f

	Intensity				
	**Chicken**	**S2**	**NL10**	**DV1-1**	**DV1-2**
	M1	M2	M3	M4	M5
Measurment 1	154104	45560	63410	78678	93681
Measurment 2	157054	46331	64372	80215	95246
Measurment 3	157009	45788	64313	79876	95191

	Calculated genome size				
	**Chicken**	**S2**	**NL10**	**DV1-1**	**DV1-2**
	M1	M2	M3	M4	M5
Total pg	2.5	0.74	1.03	1.28	1.52
Total Mb	2445	722.85	1006.06	1248.30	1486.33
Haploid Mb	1222.5	180.71	503.03	624.15	743.17
Total pg	2.5	0.74	1.02	1.28	1.52
Total Mb	2445	721.28	1002.14	1248.78	1482.78
Haploid Mb	1222.5	180.32	501.07	624.39	741.39
Total pg	2.5	0.73	1.02	1.27	1.52
Total Mb	2445	713.03	1001.50	1243.86	1482.35
Haploid Mb	1222.5	178.26	500.75	621.93	741.17

**Chromosomes**			
6S+2L=	1006.06		
6S+3L=	1248.30	L=	242.24
6S+4L=	1486.33	L=	238.04
6S+2L=	1002.14		
6S+3L=	1248.78	L=	246.64
6S+4L=	1482.78	L=	234.00
6S+2L=	1001.50		
6S+3L=	1243.86	L=	242.35
6S+4L=	1482.35	L=	238.49

Calculated sizes, Mb			
**S2**	**179.76**	±	**1.32**
**NL10**	**501.62**	±	**1.23**
**DV1**	**741.91**	±	**1.09**
**Large chr.**	**240.29**	±	**4.39**

Supplementary Figure 3 | Karyotyping and genome size measurement of the DV1 and NL10 *M. lignano* lines*.* (**a**) DV1 karyotype with 4 large chromosomes. (**b**) DV1 karyotype with 3 large chromosomes. (**c**) NL10 karyotype with 2 large chromosomes. Scale bars are 10 µm. (**d**) Separate measurements of fluorescence in DV1 and NL10 lines and chicken and *Drosophila* S2 reference cells. (**e**) Combined fluorescence measurement of all 4 genomes. (**f**) Calculation of genome sizes using chicken as a reference and *Drosophila* S2 cells as a positive control. The presence of two karyotypes in the DV1 line and the karyotype difference with the NL10 line allows to estimate the size of the large chromosome. M1-M5, gates used to calculate peak intensities of different genomes in the samples.

a

REAPR parameter	ML2	Mlig_3_7
Total length	1,040,124,789	764,424,962
Number of sequences	49,174	5,270
N50	36,723	245,921
Number of gaps	0	710
Total gap length	0	1,581,471
Error free bases	31.92%	63.95%
FCD errors within a contig	1,871	872
FCD errors over a gap	0	159
Low fragment coverage within a contig	323	136
Low fragment coverage over a gap	0	171

b

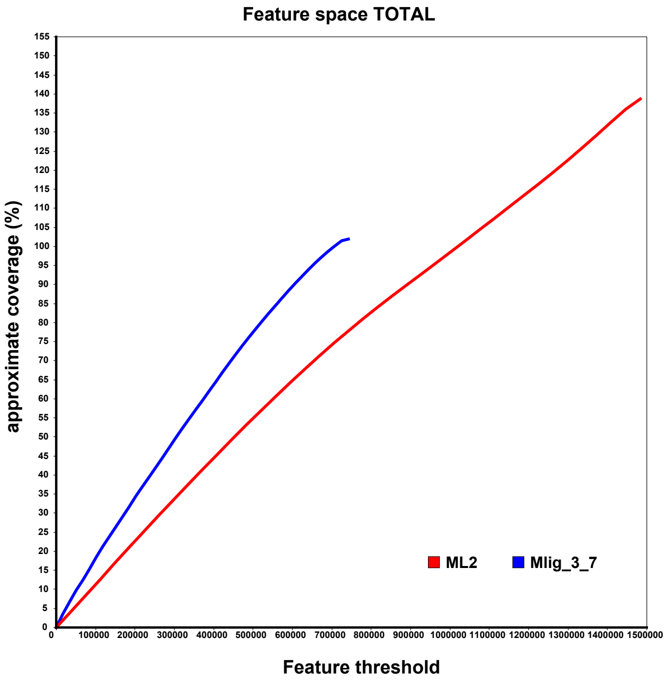

c

MLRNA150904	ML2	Mlig_3_7
non-mapped	3,080 (5.12%)	1,863 (3.10%)
same ORF	42,290 (70.27%)	45,033 (74.83%)
shorter ORF	12,365 (20.55%)	11,079 (18.41%)
longer ORF	2,445 (4.06%)	2,205 (3.66%)

Supplementary Figure 4 | Comparison of the ML2 and Mlig_3_7 genome assemblies. (**a**) REAPR evaluation. The Mlig_3_7 assembly has more error-free bases and fewer misassemblies. (**b**) FRCbam evaluation on the cumulative feature count. Steeper curves reflect better assemblies. (**c**) Mapping of the *de novo* transcriptome assembly MLRNA150904. More transcripts are mapped to the Mlig_3_7 assembly and more ORFs are preserved.

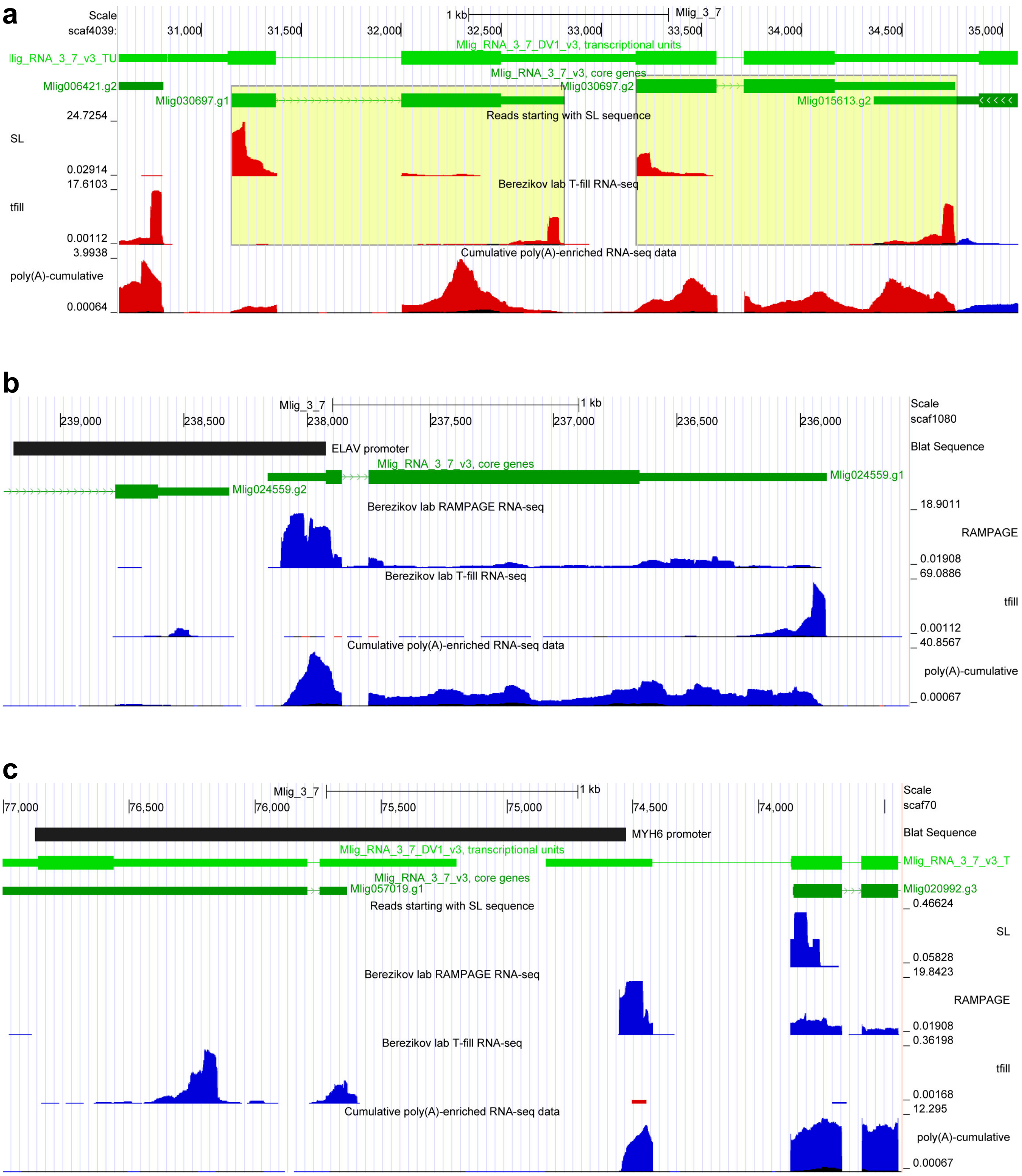

Supplementary Figure 5 | Visualisation of *M. lignano* genomic regions using the UCSC genome browser software and selection of promoters for transgenesis. (**a**) An example of a transcriptional unit with multiple trans-splicing sites. Transcriptional unit Mlig030697.1 contains two trans-spliced genes, Mlig030697.g1 and Mlig030697.g2, with 5´ and 3´ boundaries clearly defined by trans-splicing (SL) and 3´-specific (TFILL) signals, respectively. (**b**) *ELAV4* gene. RAMPAGE and TFILL signals clearly define gene boundaries. (**c**) *MYH6* gene. RAMPAGE signal is used to define the start of the gene and promoter region is selected up to the first ATG codon.

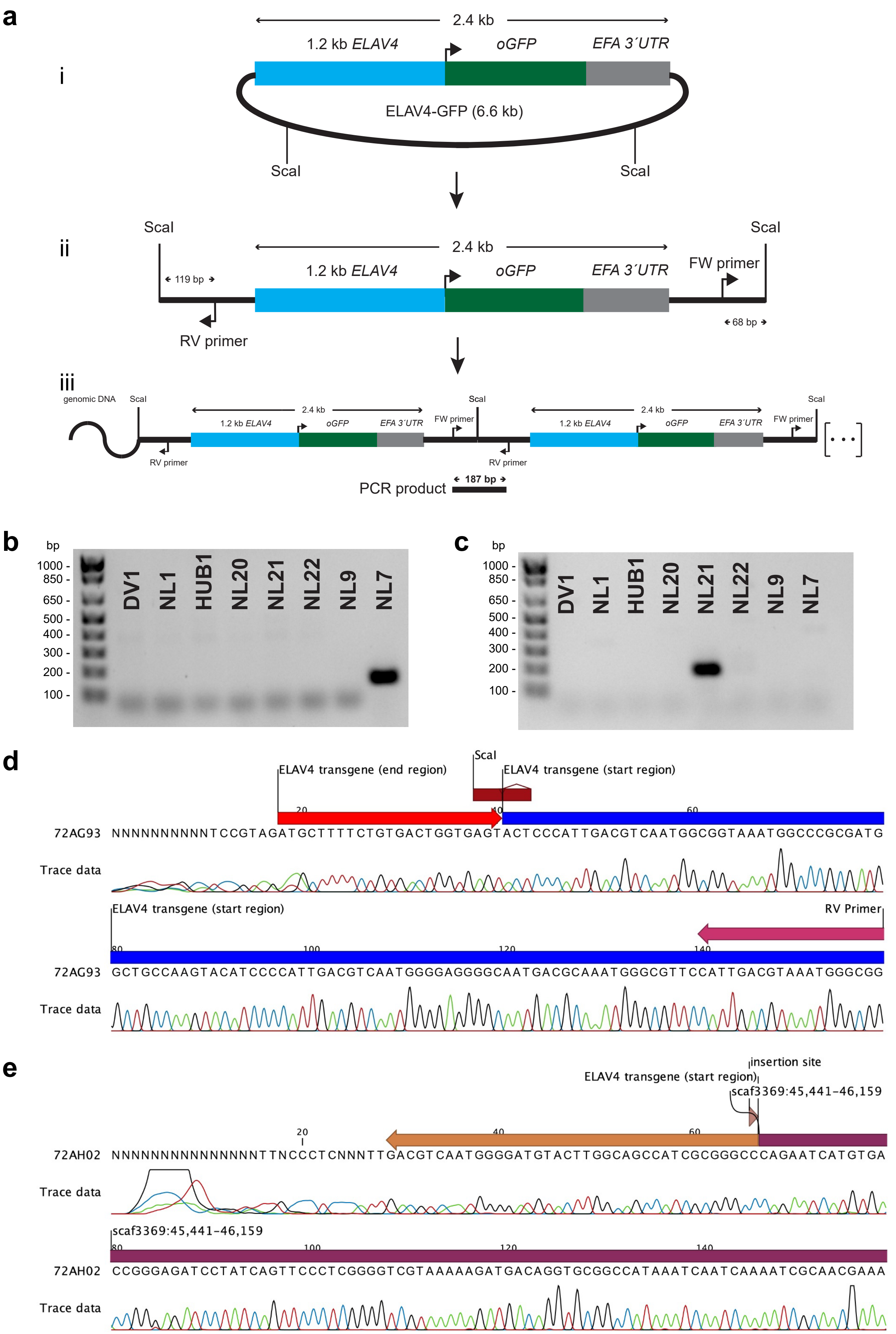

Supplementary Figure 6 | Identification of transgene integration sites. (**a**) A scheme for formation of tandem transgenes using *ELAV4::oGFP* as an example. (i) structure of the original construct; (ii) linear injected fragment; (iii) potential tandem transgene array. (**b,c**) Results of PCR from genomic DNA of different *M. lignano* lines with inverse PCR primers specific for NL7 (b) and NL21 (c) lines. In both cases PCR products of size corresponding to tandem transgene configuration are observed. (**d**) Sequencing results of the PCR product from NL21 line confirming the tandem structure of the transgene. (**e**) Sequencing results of one of the Genome Walker PCR products from NL21 line identifying integration of the transgene at position 45,440 in scaf3369.
