# Supplementary material for: Efficient transgenesis and annotated genome sequence of the regenerative flatworm model Macrostomum lignano
Source: Nat Commun. 2017 Dec 14;8:2120. doi: 10.1038/s41467-017-02214-8 (PMC5730564; doi:10.1038/s41467-017-02214-8)
Supplement: Supplementary file 3 — Description of Additional Supplementary Files [file 41467_2017_2214_MOESM3_ESM.pdf]

Description of Additional Supplementary Files

File Name: Supplementary Data 1
Description: Primers, promoters and codon-optimized sequences used for creating transgenic reporter construct.

File Name: Supplementary Movie 1
Description: Microinjection of M. lignano embryos.

File Name: Supplementary Movie 2
Description: M. lignano transgenic animal expressing GFP under control of a muscle-specific promoter MYH6.
